# The Temporal and Spatial Dynamics of Cortical Emotion Processing in Different Brain Frequencies as Assessed Using the Cluster-Based Permutation Test: An MEG Study

**DOI:** 10.3390/brainsci10060352

**Published:** 2020-06-06

**Authors:** Mina Kheirkhah, Philipp Baumbach, Lutz Leistritz, Stefan Brodoehl, Theresa Götz, Ralph Huonker, Otto W. Witte, Carsten M. Klingner

**Affiliations:** 1 Biomagnetic Center, Jena University Hospital, 07747 Jena, Germany; minakheirkhah1989@gmail.com (M.K.); stefan.brodoehl@med.uni-jena.de (S.B.); THERESA.GOETZ@med.uni-jena.de (T.G.); Ralph.Huonker@med.uni-jena.de (R.H.); 2 Department of Anesthesiology and Intensive Care Medicine, Jena University Hospital, 07747 Jena, Germany; Philipp.Baumbach@med.uni-jena.de; 3 Institute of Medical Statistics, Computer and Data Sciences, Jena University Hospital, 07740 Jena, Germany; Lutz.Leistritz@med.uni-jena.de; 4 Hans Berger Department of Neurology, Jena University Hospital, 07747 Jena, Germany; Otto.Witte@med.uni-jena.de

**Keywords:** alpha, beta, cluster-based permutation test, delta, emotion, gamma, MEG, theta

## Abstract

The processing of emotions in the human brain is an extremely complex process that extends across a large number of brain areas and various temporal processing steps. In the case of magnetoencephalography (MEG) data, various frequency bands also contribute differently. Therefore, in most studies, the analysis of emotional processing has to be limited to specific sub-aspects. Here, we demonstrated that these problems can be overcome by using a nonparametric statistical test called the cluster-based permutation test (CBPT). To the best of our knowledge, our study is the first to apply the CBPT to MEG data of brain responses to emotional stimuli. For this purpose, different emotionally impacting (pleasant and unpleasant) and neutral pictures were presented to 17 healthy subjects. The CBPT was applied to the power spectra of five brain frequencies, comparing responses to emotional versus neutral stimuli over entire MEG channels and time intervals within 1500 ms post-stimulus. Our results showed significant clusters in different frequency bands, and agreed well with many previous emotion studies. However, the use of the CBPT allowed us to easily include large numbers of MEG channels, wide frequency, and long time-ranges in one study, which is a more reliable alternative to other studies that consider only specific sub-aspects.

## 1. Introduction

The generation of emotions in the human brain is an extremely complex process, and the statistical analysis of this process is an important focus in neuroscience. Among the non-invasive brain measurement techniques for measuring brain reactions to emotional stimuli, the use of MEG is very popular because of its high temporal and spatial resolution. Since MEG data are sampled over multiple channels and time points, it results in an extremely large amount of data, necessitating extensive computations [1,2]. One approach to overcome this problem is by use of the cluster-based permutation test (CBPT [3]) which results in both high Type II (power) and nominal Type I (false positive) error rates [2,4]. In brief, the CBPT assumes that the signals are clustered along the dimensions of data (i.e., frequency × space × time). This assumption is based on the fact that when a cortical source becomes active, adjacent sensors show similar patterns and correlated activity, and this activity has a temporal duration at different frequencies. That means the CBPT makes no inferences over one specific time point at one specific sensor at one specific frequency, but instead compares the cluster structure of the observed data for different conditions to the cluster structure constructed under the null hypothesis [1,2,5,6]. The null hypothesis is defined as the difference between the two conditions being zero. In practice, when using nonparametric statistical tests to compare two conditions, the trials of the two conditions are randomly drawn and are placed into two subsets (random partition), and the test statistics are calculated based on these two random partitions. After repeating this procedure for a large number of times, a histogram of test statistics is constructed, which is called the permutation distribution. The significant clusters, typically via the approach of Maris and Ostenveld, are the clusters with the permutation distribution of the maximum within-cluster summed *t*-values [3]. Since nonparametric statistical tests can process data from any distribution (e.g., binominal, Bernoulli) and test statistics based on any statistical inference (e.g., *t*-test, *z*-test), they are a very generalizable alternative to traditional parametric statistical frameworks such as ANOVAs [4].

To process brain reactions to emotional stimuli, different approaches have been implemented in various studies. For instance, one MEG study examined the brain responses of children when viewing their own parent–child interactions and calculated statistics at the source-level using the analysis of functional neuroimages (AFNI) toolbox [7]. Another MEG study by Styliadis investigated the source distribution of gamma band activity when viewing images with different arousal and valence levels, using a beamformer and a sliding window method [8]. Jabbi and colleagues collected MEG and fMRI measurements of the same participants when viewing videos of either emotional facial expressions or emotionally neutral facial movements [9]. They performed a source-level analysis and implemented a robust false discovery rate (FDR) method to correct the multiple comparisons in the space–time–frequency domain. Grootswagers and colleagues investigated the analysis of brain responses to image stimuli measured by MEG using multivariate pattern analysis (MVPA) [10]. Thus, it appears that different studies have applied different approaches to analyze brain responses to emotional stimuli, highlighting the complexity and multiple aspects of emotion processing. However, when considering the CBPT method, although many studies have applied this method to electroencephalographic (EEG) and MEG data (e.g., References [2,3,11,12,13,14]), to the best of our knowledge there have been only two studies [15,16] that used this method in the analysis of brain responses to emotional versus neutral stimuli. In one of these studies, by Jessen and Kotz [15], the authors used the CBPT on EEG data at the sensor level, and examined only the alpha and beta bands during the 250–1000 ms after stimulus onset. In the study by Bublatzky [16], the author performed a cluster-based analysis at the source level on the brain responses to emotional facial stimuli measured by MEG, and they did not evaluate their results on different frequency bands. Although both studies reported very valuable results, the inclusion of more frequency and time domains in these experiments could help to obtain more information about the processing of emotions. In addition, it could reduce the variation in experimental designs, recording strategies, and analysis procedures compared to the consideration of different frequencies and time ranges in different studies to date [17]. Therefore, to evaluate the relationship between different emotions and brain oscillations, the consideration of a wide range of frequencies and time intervals in one experiment is advantageous. Taking into account this advantage, we decided to apply the CBPT in the analysis of brain responses to emotional versus neutral stimuli, considering frequency ranges from 1 to 45 Hz and time intervals within 1500 ms post-stimulus. To define the conditions of the CBPT, we decided to focus on the arousal factor (pleasant versus neutral; unpleasant versus neutral) to investigate the temporal and spatial dynamics of cortical responses to emotional versus neutral stimuli at different frequencies. To measure the brain responses, we used MEG, because many studies have reported highly accurate MEG results [18,19,20,21], or more accurate results compared to the use of EEG [22] in emotion processing. Since no study, to the best of our knowledge, has considered the CBPT approach in brain responses to emotional stimuli at the sensor level in MEG measurement, this study will provide a clear path for future studies in this field. This study also attempted to verify the findings of many previous studies (e.g., References [23,24,25,26,27]) in one study, covering a wide range of frequencies, time, and MEG channels using the CBPT.

## 2. Materials and Methods

In order to investigate the temporal dynamics of emotional reactions in the human brain at different brain frequencies using the CBPT, we applied the methodology described below.

### 2.1. Subjects

A total of 17 healthy subjects (11 females; aged 19–33 years; mean age 26.9 years) participated in this study. All subjects had normal or corrected-to-normal vision, and no history of neurological or psychiatric disorders. The details of the study were approved by the local ethics committee of Jena University Hospital (4415-04/15) and all subjects gave their written informed consent.

### 2.2. Stimuli and Design

**Figure 1:**
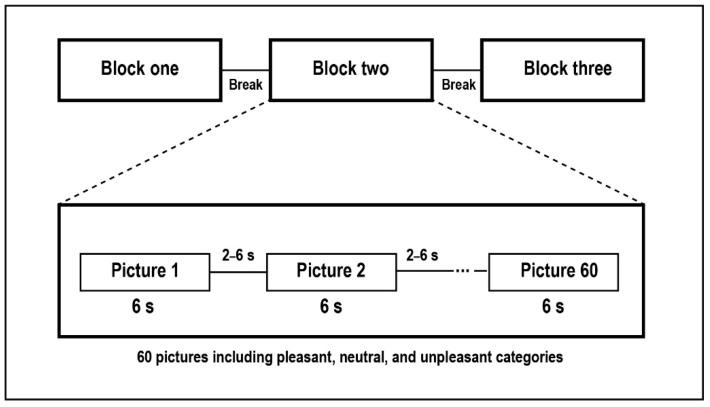
Schematic view of the presentation of pictures in this study.

The stimuli were selected from the International Affective Picture System (IAPS [28]) consisting of 180 color pictures in three categories (60 pictures each): pleasant, neutral, and unpleasant. A broad range of content was covered in the selected IAPS pictures. For example, sports scenes and happy families were selected for pleasant content, attack scenes and mutilated bodies for unpleasant content, and household objects and neutral faces comprised the neutral pictures. The presentation of the pictures was divided into three blocks consisting of 20 pictures from each category in a pseudo-randomized order, and each block was followed by a short break that allowed the subject to relax. Each picture was presented for 6000 ms, with a maximum size of 30.9 cm × 41.5 cm (see Figure 1), on a screen placed about 105 cm in front of the subject with a visual angle of 16.5° × 21.5°. Two successive trials were separated by randomized intervals of between 2000 and 6000 ms. The luminance of the pictures was measured using the MATLAB Lab color-space toolbox. The mean luminance of the selected pictures in each category of pleasant, unpleasant, and neutral was matched. The image contrast was measured using ImageJ as the standard deviation of the mean pixel values from grayscale images, and the pictures in each category were matched in contrast.

During the viewing of the pictures, subjects were asked to avoid movement as much as possible. The entire measuring procedure was performed in a sound-sheltered and magnetically shielded room in the Bio-Magnetic Center of Jena University Hospital, and lasted about 45 min.

### 2.3. Data Acquisition and Analysis

MEG data were recorded using a 306 channel helmet-shaped Elekta Neuromag MEG system (Vectorview, Elekta Neuromag Oy, Helsinki, Finland) comprising 204 gradiometers and 102 magnetometers. Before the MEG measurement, the Cartesian coordinate system of each subject’s scalp was defined using a 3D digitizer (3SPACE FASTRAK, Polhemus Inc., Colchester, VT, USA). MEG data were digitized to 24 bit at a sampling rate of 1 kHz. To quantify the same MEG channel positions for all subjects, we applied MaxFilter Version 2.0.21 (Elekta Neuromag Oy, Helsinki, Finland) to raw data using the signal-space separation (SSS) method [29], with sensor-level data alignment across all subjects to a reference subject. We used only the data of the 102 magnetometers, which had higher signal-to-noise ratios (SNR) than gradiometers in our experiment. Another reason for this is that after performing the SSS, magnetometer and gradiometer datasets produce highly similar results, since SSS removes device-dependent external disturbances and reconstructs the sensor data from a single set of SSS components [30,31,32]. Moreover, taking only magnetometer data using an SSS method is common in MEG studies of emotion-related topics (e.g., Reference [32]). Data were downsampled to 250 Hz. An electrooculogram (EOG) was recorded to facilitate the detection of eye activity. For this purpose, two bipolar electrodes were placed below and above the left eye to record vertical eye movements (eyeblink), and two additional bipolar electrodes at the outer angle of the left and right eye for horizontal eye movements (e.g., eye rolls). In order to estimate the artefacts caused by the magnetic fields of the heartbeat, one electrocardiogram (ECG) electrode was attached to the right collarbone and one to the left thorax (below the caudal costal arch). Eye artefacts and artefacts caused by magnetic fields of the heartbeat were removed by applying independent component analysis (ICA; runica) with 30 dimensions. Two ECG components and two or three EOG components for each subject were removed. In order to detect and remove trials with excessive movement artefacts, visual detection was used. Finally, 45 to 55 trials remained for each stimulus category per subject.

After artefact removal, time–frequency representations (TFRs) with a frequency range of 1–45 Hz were calculated using the function ft_freqanalysis in the FieldTrip toolbox. TFRs were computed based on the multi-taper-convolution (mtmconvol) method with a Hanning taper. The mtmconvol method implements a sliding window FFT and the Hanning taper has the advantage of fully limiting the time dispersion to the time window of interest [33] which was defined from 500 ms pre-stimulus to 1500 ms post-stimulus in our study. A relative baseline correction was performed to the 500 ms pre-stimulus onset. Epochs were extracted from 500 ms pre-stimulus to 1500 ms post-stimulus onset with a baseline correction of 500 ms pre-stimulus. The entire analysis was performed using MATLAB 9.3.0 (Mathworks, Natick, MA, USA) and FieldTrip toolbox 2018 [1].

### 2.4. Time Intervals of Interest

**Figure 2:**
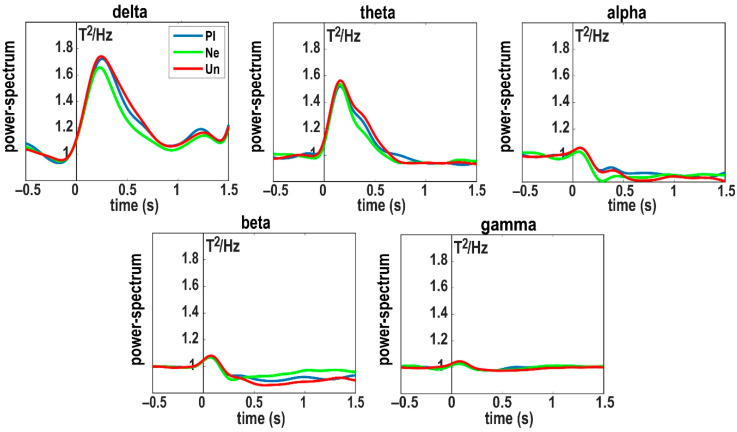
Group-related power spectra for each picture category and each frequency band. Power spectra for three picture categories (pleasant (Pl): blue; unpleasant (Un): red; neutral (Ne): green) and five frequency bands were averaged over all 102 MEG channels and all 17 subjects.

We averaged the power spectra in each frequency band across all channels and all subjects to define time intervals of interests based on the most pronounced peaks in group-related power-spectra in each frequency band (see Figure 2). It is worth noting that these group-related power spectra were not used in the CBPT and were only intended to specify time intervals. As shown in Figure 2, the most pronounced peaks of the group-related power spectra in each frequency bands were as follows: delta: 200–300 ms; theta: 150–200 ms; alpha: 100–150 ms and 290–430 ms; beta: 80–250 ms; gamma: 0–120 ms. In addition to considering these time intervals, we also included the later time interval of 500–1000 ms based on the time intervals used in the study by Jessen and Kotz [15] which applied the CBPT on EEG data of brain responses to emotional and neutral stimuli. Moreover, we also examined our results within the 1000–1500 ms window to see whether there was useful information after 1 s.

### 2.5. Applying the Cluster-Based Permutation Test (CBPT)

To determine the temporal dynamics of brain responses to emotional (pleasant and unpleasant) versus neutral stimuli at different frequency bands, we applied the CBPT as implemented in FieldTrip [1,3,34]. This was performed based on the specific time intervals of interest, which were defined in the previous section, and all 102 sensors for each frequency band. Clusters had to extend over at least three adjacent magnetometers as a constraint. If the calculated *p*-value was less than the critical alpha level of 0.050, then the clusters of differences between emotional and neutral conditions were considered significant [34]. Although the Monte Carlo *p*-values were calculated on the basis of 1000 random partitions for each frequency individually, we reported the results based on individual *p*-values. We applied the Benjamini & Hochberg/Yekutieli false discovery rate [35,36,37] to avoid the multiple comparison problem and to adjust the cluster *p*-values calculated on the basis of different comparisons (pleasant vs. neutral; unpleasant vs. neutral) at different time intervals of interest for each frequency of bands. This was implemented using the source code provided by David M. Groppe [38].

It should be noted that the term “positive cluster” in this study refers to a cluster that showed higher power for emotional stimuli than for neutral stimuli, and the term “negative cluster” means a cluster that showed higher power for neutral stimuli than for emotional stimuli.

To ensure that the results of clusters (selected time intervals and channels) were reliable for the whole frequencies in each frequency band, we applied a classification algorithm, logistic regression with the most popular penalty, the Least Absolute Shrinkage and Selection Operator (LASSO; [39]). This also helped to find the best channels and time intervals selected by CBPT to classify brain responses to emotional versus neutral stimuli. We defined features based on the average power spectrum in each frequency band, with channels and time intervals selected by the CBPT. Classification performances were evaluated on the basis of their accuracy, defined as the ratio of correctly classified samples to their total number. The accuracies were assessed by performing 1000 17-fold stratified cross-validations, and 95% confidence intervals are reported. Further details of the classification method used in this study are provided in the Supplementary Materials.

## 3. Results

In this section, we present the results of the significant clusters identified for different frequencies. To avoid stating many *p*-values related to each frequency, we have instead specified the largest *p*-value in parentheses. We also present the classification performances related to each cluster based on accuracies with 95% confidence intervals.

It should be noted that the brain areas corresponding to detected clusters were defined based on Elekta Neuromag channel positions (see Supplementary Figure S1). Please also note that the time windows shown in the images are based on the input time (time intervals of interest) into the CBPT, but the time intervals selected by significant clusters are written in the text.

### 3.1. Delta Band (1–4 Hz)

**Figure 3:**
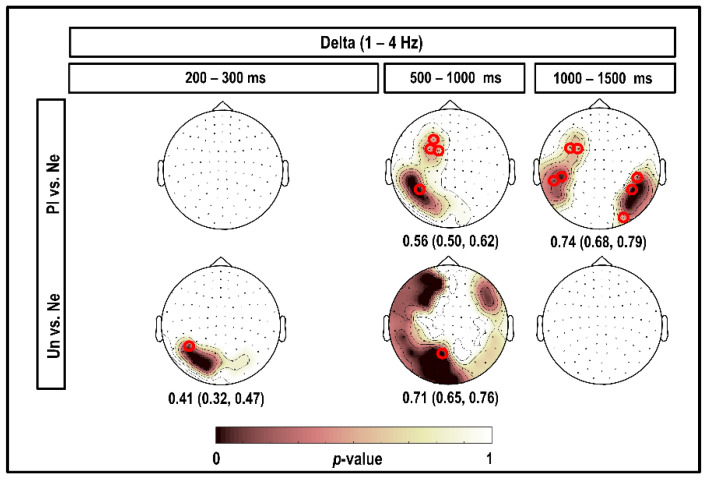
Topography of the significant clusters in the delta band according to the CBPT, and corresponding classification performances. Channels that were part of the clusters for the comparison between brain responses to emotional (pleasant (Pl) and unpleasant (Un)) versus neutral (Ne) stimuli in the delta band are highlighted. Numerical values under the topo-plots represent medians as well as 95% confidence intervals for classification accuracies, considering the highlighted channels selected by CBPT and corresponding time intervals. The small red circles indicate the channels selected by the classifier, which were more fit than other channels to classify brain responses to emotional versus neutral stimuli in each cluster.

Figure 3 depicts the results of using the CBPT in the delta band, considering all magnetometers and time intervals within 1500 ms post-stimulus. As illustrated in this figure, a positive cluster was found in the left occipital channels within 200–300 ms of viewing unpleasant (significant to 1 Hz; *p* = 0.030) stimuli compared to neutral stimuli. A very large positive cluster of significant differences was found within 500–620 ms in the left temporal–occipital and left prefrontal channels comparing responses to pleasant versus neutral stimuli (significant to 1–2 Hz and 4 Hz, respectively; *p* < 0.050). In the same time window, 500–620 ms, we found a positive cluster covering the right prefrontal, right temporal, right temporal–occipital, occipital, left temporal–occipital, left temporal, and left prefrontal channels, comparing brain responses to unpleasant versus neutral stimuli (significant to 3 and 4 Hz; *p* = 0.010). At 1080–1250 ms, we observed a positive cluster showing a higher delta power for brain responses to pleasant versus neutral stimuli (significant at 1–2 Hz; *p* = 0.030), and we did not find any significant clusters when comparing responses to unpleasant and neutral stimuli.

The classification performances, based on 95% confidence intervals of accuracies, are also shown in Figure 3. As can be seen, classification based on the channels selected by CBPT at 200–300 ms resulted in low accuracies and was not reliable for the whole 1–4 Hz. The highest accuracies of 0.74 (95% CI (0.68; 0.79)) and 0.71 (95% CI (0.65; 0.76)) were obtained, respectively, for the comparison of delta power in response to pleasant versus neutral at 1080–1250 ms and in response to unpleasant versus neutral at 500–620 ms.

### 3.2. Theta Band (5–8 Hz)

**Figure 4:**
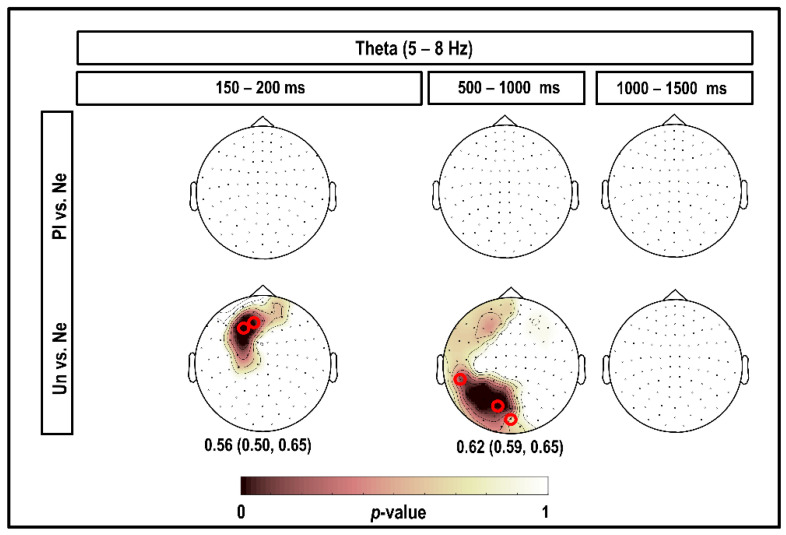
Topography of the significant clusters in the theta band using the CBPT and corresponding classification performances. Channels that were part of the clusters for the comparison between brain responses to emotional (pleasant (Pl) and unpleasant (Un)) versus neutral (Ne) stimuli in the theta band are highlighted. Numerical values under the topo-plots represent medians as well as 95% confidence intervals for classification accuracies, considering the highlighted channels selected by CBPT and corresponding time intervals. The small red circles indicate the channels selected by the classifier, which were better suited than other channels to classify brain responses to emotional versus neutral stimuli in each cluster.

Figure 4 depicts the results of the CBPT in the theta band, considering all magnetometers and time intervals within the 1500 ms post-stimulus. As shown in this figure, a very focal positive cluster of significant differences was observed at the left parietal and prefrontal magnetometers, indicating stronger theta power in response to unpleasant than neutral stimuli, extending over 150–200 ms (significant at 6–8 Hz; *p* = 0.036). In the same time interval, 150–200 ms, no significant cluster was found when comparing brain responses to pleasant versus neutral stimuli. Moreover, a positive cluster in the left occipital, left temporal, and left prefrontal channels with stronger theta power in response to unpleasant compared to neutral stimuli was observed at 500–600 ms (significant at 5 Hz; *p* = 0.021). We found no significant clusters at 1350–1500 ms.

As can be seen in Figure 4, all the classification performances were significant, showing that the results were reliable for the whole range of frequencies in the theta band. The highest accuracy of 0.62 (95% CI (0.59; 0.65)) was obtained considering theta power in response to unpleasant versus neutral at 500–600 ms.

### 3.3. Alpha Band (9–13 Hz)

**Figure 5:**
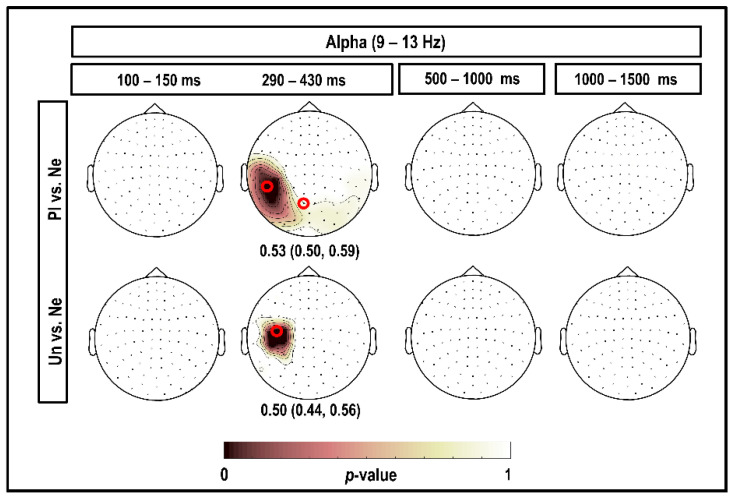
Topography of the significant clusters in the alpha band using the CBPT and corresponding classification performances. Channels that were part of the clusters for the comparison between brain responses to emotional (pleasant (Pl) and unpleasant (Un)) versus neutral (Ne) stimuli in the alpha band are highlighted. Numerical values under the topo-plots represent medians as well as 95% confidence intervals for classification accuracies, considering the highlighted channels selected by CBPT and corresponding time intervals. The small red circles indicate the channels selected by the classifier, which were better suited than other channels to classify brain responses to emotional versus neutral stimuli in each cluster.

Figure 5 depicts the results of the CBPT in the alpha band, considering all magnetometers and time intervals within 1500 ms post-stimulus. Our results showed no significant clusters when considering the time interval of 100–150 ms. When comparing brain responses to pleasant versus neutral stimuli, we observed stronger alpha power for pleasant stimuli in the whole occipital, temporal–occipital, and parietal–occipital channels, with more focus in the left side, at 290–430 ms (significant at 9–13 Hz; *p* = 0.050). For comparison between brain responses to unpleasant versus neutral stimuli in the same time window, 290–430 ms, we observed a high alpha power in the left parietal channels (significant at 13 Hz; *p* = 0.050). We found no significant clusters for the comparison between alpha power of brain responses to emotional versus neutral stimuli after 500 ms post-stimulus.

As can be seen in Figure 5, all the classification performances were significant. The accuracies of 0.53 (95% CI (0.50; 0.59)) and 0.50 (95% CI (0.44; 0.56)) were obtained by considering alpha power in response to pleasant versus neutral and unpleasant versus neutral, respectively, at 290–430 ms.

### 3.4. Beta Band (14–25 Hz)

**Figure 6:**
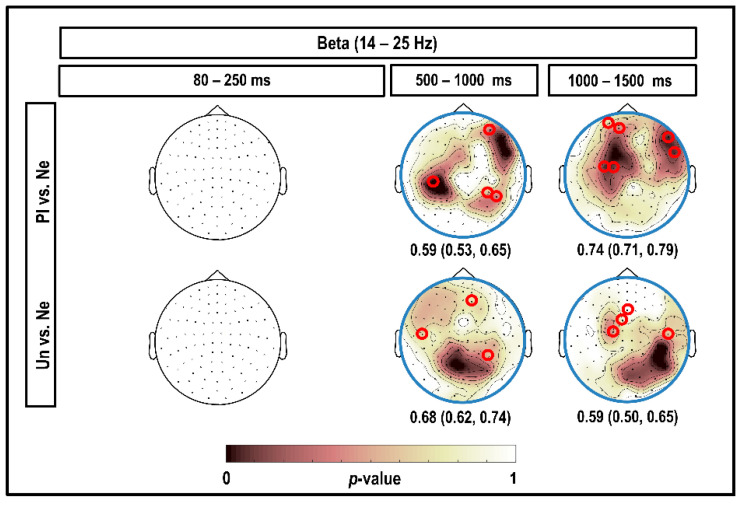
Topography of the significant clusters in the beta band using the CBPT and corresponding classification performances. Channels that were part of the clusters for the comparison between brain responses to emotional (pleasant (Pl) and unpleasant (Un)) versus neutral (Ne) stimuli in the beta band are highlighted. The topo-plots outlined in blue indicate the negative clusters with higher beta power for neutral stimuli compared to emotional stimuli. Numerical values under the topo-plots represent medians as well as 95% confidence intervals for classification accuracies, considering the highlighted channels selected by CBPT and corresponding time intervals. The small red circles indicate the channels selected by the classifier, which were better suited than other channels to classify brain responses to emotional versus neutral stimuli in each cluster.

Figure 6 depicts the results of the CBPT in the beta band, considering all magnetometers and time intervals within the 1500 ms post-stimulus. Our results showed no significant clusters for comparison between the emotional and neutral stimuli at 80–250 ms post-stimulus. A negative cluster with higher beta power was observed for brain responses to neutral compared to pleasant stimuli at 800–1000 ms in the right prefrontal, left parietal, and right parietal–occipital channels (significant at 16–18 Hz; *p* < 0.050). Left parietal, left prefrontal, right prefrontal, and occipital showed a very large negative cluster indicating high beta power for brain responses to neutral stimuli at 1200–1300 ms compared to pleasant stimuli (significant at 16–19 Hz; *p* < 0.050). We also found a negative cluster showing higher beta power for brain responses to neutral stimuli than unpleasant stimuli at 800–1000 ms over parietal, prefrontal and occipital channels, with more effect on parietal–occipital channels (significant at 14–19 Hz; *p* < 0.050). In addition, at 1000–1500 ms, we found a negative cluster with higher beta power for brain responses to neutral versus unpleasant stimuli in the prefrontal and parietal channels, with more effect in the right parietal–occipital channels (significant in 14–19 Hz; *p* < 0.050).

As can be seen in Figure 6, all the classification performances were significant. The highest accuracy comparing beta power in response to pleasant versus neutral stimuli was obtained at 1200–1300 ms, with an accuracy of 0.74 (95% CI (0.71; 0.79)). The highest accuracy comparing beta power in response to unpleasant versus neutral stimuli was obtained at 800–1000 ms, with an accuracy of 0.68 (95% CI (0.62; 0.74)).

### 3.5. Gamma Band (26–45 Hz)

Considering gamma power, we found no significant clusters during time intervals within 1500 ms post-stimulus.

## 4. Discussion

In this study, we investigated the temporal and spatial dynamics of the human brain reactions to emotional stimuli at different frequencies using the CBPT. We measured the brain responses of 17 subjects to three categories of picture stimuli (pleasant, neutral, and unpleasant) using MEG. Our results revealed significant clusters, showing high differences between the processing of emotional versus neutral stimuli at different MEG channels, frequencies, and time-ranges. Since the clusters were sometimes significant only for some frequencies in each frequency band, we evaluated the channels and time selected by the clusters over the average of all frequencies in each band using classification methods. In the following sections, the results of significant clusters, which were also significant in the classifications, are discussed separately in different brain frequency bands.

### 4.1. Delta

In the first 1000 ms after viewing picture stimuli, we found higher delta power in brain responses to emotional compared to neutral stimuli, with more focus in the left temporal and parietal channels (as the red circles and highlighted areas in Figure 3 show). Since the emotional (pleasant and unpleasant) picture stimuli in this study produced higher arousal levels than the neutral ones (see Supplementary Figure S2, and Table S1), our results were consistent with the results of many studies, which have indicated that high-arousal pictures produce greater event-related delta synchronization than low-arousal pictures [24,40,41,42]. This finding was also comparable with the results of a study by Sakihara and colleagues [27], which showed that a familiar face that elicits emotions produces higher delta responses over the parietal and left temporal regions than an unfamiliar face.

### 4.2. Theta

Our results showed that the theta power within 600 ms of viewing unpleasant pictures was higher than that after viewing neutral pictures in the frontal and occipital regions (as the red circles and highlighted areas in Figure 4 show). This was in line with the findings of previous studies showing that arousal discrimination is associated with increased theta power upon the presentation of IAPS pictures [40,43,44]. I has also been reported that theta oscillations are the dominant rhythms of the frontal cortex [45]. Moreover, many studies have reported that during the presentation of emotional paradigms, the occipital region produces synchronized theta oscillations [23,43,46,47]. In addition, Aftanas and colleagues demonstrated that only affective valence stimuli can generate theta power in the posterior brain regions, and not neutral stimuli [46]. Sun and colleagues revealed that viewing negative IAPS pictures triggers higher theta power in the posterior regions than viewing neutral IAPS pictures at 150–200 ms post-stimulus [48]. This result was also consistent with many studies that presented facial expression stimuli [23,47].

### 4.3. Alpha

Our results considering alpha power showed that comparing either pleasant or unpleasant versus neutral, significant effects on the left parietal area were apparent. This was in line with the results of a study by Güntekin and Basar that reported that viewing angry faces increased the amplitudes of alpha power (9–13 Hz) over the parietal and temporal areas [49]. The effects of emotional stimuli on alpha power are still less clear than at other frequencies, and research on the alpha band has focused more on frontal alpha asymmetry or alpha-event-related desynchronization [50].

### 4.4. Beta

The investigation of the effects of emotional stimuli on beta frequencies in our study demonstrated beta suppression upon viewing emotional stimuli compared to neutral stimuli in the posterior, prefrontal, and central magnetometers at 800–1200 ms post-stimulus (as the red circles and highlighted areas in Figure 6 show). This finding was in line with the results of Jessen and Kotz, who used the CBPT in EEG data and found beta suppression for brain responses to fear versus neutral visual stimuli in the posterior regions at 750–1000 ms [15]. In our results, the only frequency band that showed power suppression for emotional stimuli compared to neutral stimuli was the beta band. Engel and Fries reported associations between beta-band synchronization and state maintenance, so the suppression of beta power during emotional states in Jessen’s study and ours could indicate that the subject becomes adapted to the observed emotional stimuli after 800 ms [15,51]. However, in contrast to our results, Miskovic and colleagues also found an increased beta response for pleasant and unpleasant picture stimuli compared to neutral pictures in a wide time range when free viewing the images [26].

### 4.5. Gamma

We did not find any significant clusters when considering gamma power in time intervals within the 1500 ms post-stimulus. However, based on many emotion studies that found different gamma power in response to emotional stimuli versus neutral stimuli (e.g., References [52,53]) we expected that gamma power in our study would show significant clusters. This result may be a sign that CBPT is not a good choice for high frequencies like gamma. However, to our knowledge, there have been no studies to date that have analyzed gamma oscillations in response to emotions using CBPT, so we were unable to evaluate our results in comparison to others’.

It is worth noting that in studies considering image stimuli, differences between brain responses may also be related to different low-level properties of images. Existing differences in low-level properties of visual mental imagery like different luminance values or contrast modulate the stimulus-evoked brain responses [54,55,56]. For example, images with larger size and higher contrast lead to a higher amplitude of the P1 and N1 components in event-related potentials (ERPs), which represent the early cognitive processes. However, these low-level properties can only modulate the early brain responses (i.e., N1 and P1), and the later responses are independent of the physical properties of the stimulus [55,56]. Accordingly, in our study, where we were comparing brain responses to emotional versus neutral stimuli within the 1500 ms post-stimulus, standardization of low-level properties of pictures was important and, as we mentioned earlier, they were matched between the pictures of emotional and neutral categories. Thus these properties could not have contributed to the differences we found in our comparisons.

## 5. Future Work

We suggest some interesting points for further studies in this area. First, we performed CBPT on MEG data at the sensor level, and performing CBPT at the source level considering different frequency bands might also provide very interesting results in future studies. Second, we did not consider the hemispheric dominance of subjects, which could have an impact on emotion processing, and taking this into consideration might help to make a more precise analysis of the emotions.

## 6. Conclusions

This study provides a comprehensive investigation of the brain reactions to emotional versus neutral stimuli, taking into account MEG channels covering the responses of the entire cortex, a wide frequency range, and a range of temporal intervals, and considering all the parameters that were easily possible with the CBPT. To the best of our knowledge, our study was the first to apply the CBPT to MEG data of brain responses to emotional stimuli at the sensor level, and provides a direction for future research in this field.

## Supplementary Materials

The following are available online at https://www.mdpi.com/2076-3425/10/6/352/s1. Figure S1: Presentation of the MEG sensor grouping in five brain areas, Figure S2: Mean rating of arousal and valence over subjects for each picture category. Table S1: Results of comparing mean ratings of subjects for each picture category in this study.

## References

[1] Oostenveld R., Fries P., Maris E., Schoffelen J.-M. (2010). FieldTrip: Open source software for advanced analysis of MEG, EEG, and invasive electrophysiological data. Comput. Intell. Neurosci..

[2] Sassenhagen J., Draschkow D. (2019). Cluster-based permutation tests of MEG/EEG data do not establish significance of effect latency or location. Psychophysiology.

[3] Maris E., Oostenveld R. (2007). Nonparametric statistical testing of EEG- and MEG-data. J. Neurosci. Methods.

[4] Frömer R., Maier M., Rahman R.A. (2018). Group-level EEG-processing pipeline for flexible single trial-based analyses including linear mixed models. Front. Mol. Neurosci..

[5] Maris E. (2004). Randomization tests for ERP topographies and whole spatiotemporal data matrices. Psychophysiology.

[6] Maris E. (2011). Statistical testing in electrophysiological studies. Psychophysiology.

[7] Pratt M., Goldstein A., Feldman R. (2018). Child brain exhibits a multi-rhythmic response to attachment cues. Soc. Cogn. Affect. Neurosci..

[8] Styliadis C., Ioannides A.A., Bamidis P.D., Papadelis C. (2018). Mapping the spatiotemporal evolution of emotional processing: An MEG study across arousal and valence dimensions. Front. Hum. Neurosci..

[9] Jabbi M., Kohn P.D., Nash T., Ianni A., Coutlee C., Holroyd T., Carver F.W., Chen Q., Cropp B., Kippenhan J.S. (2014). Convergent BOLD and beta-band activity in superior temporal sulcus and frontolimbic circuitry underpins human emotion cognition. Cereb. Cortex.

[10] Grootswagers T., Kennedy B.L., Most S.B., Carlson T.A. (2017). Neural signatures of dynamic emotion constructs in the human brain. Neuropsychologia.

[11] Huang G., Zhang Z. Improving sensitivity of cluster-based permutation test for EEG/MEG data. Proceedings of the 2017 8th International IEEE/EMBS Conference on Neural Engineering (NER).

[12] Pernet C., Latinus M., Nichols T., Rousselet G. (2014). Cluster-based computational methods for mass univariate analyses of event-related brain potentials/fields: A simulation study. J. Neurosci. Methods.

[13] Piai V., Dahlslätt K., Maris E. (2014). Statistically comparing EEG/MEG waveforms through successive significant univariate tests: How bad can it be?. Psychophysiology.

[14] Urigüen J.A., Garcia-Zapirain B., Artieda J., Iriarte J., Valencia M. (2017). Comparison of background EEG activity of different groups of patients with idiopathic epilepsy using Shannon spectral entropy and cluster-based permutation statistical testing. PLoS ONE.

[15] Jessen S., Kotz S. (2011). The temporal dynamics of processing emotions from vocal, facial, and bodily expressions. NeuroImage.

[16] Bublatzky F., Kavcıoğlu F., Guerra P., Doll S., Junghöfer M. (2020). Contextual information resolves uncertainty about ambiguous facial emotions: Behavioral and magnetoencephalographic correlates. NeuroImage.

[17] Esslen M., Pascual-Marqui R.D., Hell D., Kochi K., Lehmann D. (2004). Brain areas and time course of emotional processing. NeuroImage.

[18] Giorgetta C., Grecucci A., Bonini N., Coricelli G., Demarchi G., Braun C., Sanfey A.G. (2013). Waves of regret: A meg study of emotion and decision-making. Neuropsychologia.

[19] Kheirkhah M., Brodoehl S., Leistritz L., Götz T., Baumbach P., Huonker R., Witte O.W., Volk G.F., Guntinas-Lichius O., Klingner C. (2020). Abnormal emotional processing and emotional experience in patients with peripheral facial nerve paralysis: An MEG study. Brain Sci..

[20] Lu Q., Li H., Luo G., Wang Y., Tang H., Han L., Yao Z. (2012). Impaired prefrontal-amygdala effective connectivity is responsible for the dysfunction of emotion process in major depressive disorder: A dynamic causal modeling study on MEG. Neurosci. Lett..

[21] Peyk P., Schupp H.T., Elbert T., Junghöfer M. (2008). Emotion processing in the visual brain: A MEG analysis. Brain Topogr..

[22] Abadi M.K., Subramanian R., Kia S.M., Avesani P., Patras I., Sebe N. (2015). DECAF: MEG-based multimodal database for decoding affective physiological responses. IEEE Trans. Affect. Comput..

[23] Güntekin B., Başar E. (2009). Facial affect manifested by multiple oscillations. Int. J. Psychophysiol..

[24] Güntekin B., Başar E. (2014). A review of brain oscillations in perception of faces and emotional pictures. Neuropsychologia.

[25] Keil A., Müller M.M., Gruber T., Wienbruch C., Stolarova M., Elbert T. (2001). Effects of emotional arousal in the cerebral hemispheres: A study of oscillatory brain activity and event-related potentials. Clin. Neurophysiol..

[26] Miskovic V., Schmidt L.A. (2010). Cross-regional cortical synchronization during affective image viewing. Brain Res..

[27] Sakihara K., Gunji A., Furushima W., Inagaki M. (2012). Event-related oscillations in structural and semantic encoding of faces. Clin. Neurophysiol..

[28] Lang P.J., Bradley M.M., Cuthbert B.N. (1997). International Affective Picture System (IAPS): Technical Manual and Affective Ratings.

[29] Taulu S., Simola J. (2006). Spatiotemporal signal space separation method for rejecting nearby interference in MEG measurements. Phys. Med. Biol..

[30] Taulu S., Simola J., Kajola M. (2005). Applications of the signal space separation method. IEEE Trans. Signal. Process..

[31] Garces P., López-Sanz D., Maestú F., Pereda E. (2017). Choice of magnetometers and gradiometers after signal space separation. Sensors.

[32] García J.P., Garcés P., Del Río D., Maestú F. (2017). Tracking the effect of emotional distraction in working memory brain networks: Evidence from an MEG study. Psychophysiology.

[33] Popov T., Oostenveld R., Schoffelen J.M. (2018). FieldTrip made easy: An analysis protocol for group analysis of the auditory steady state brain response in time, frequency, and space. Front. Mol. Neurosci..

[34] FieldTriptoolbox Cluster-Based Permutation Tests on Time-Frequency Data. http://www.fieldtriptoolbox.org/tutorial/cluster_permutation_freq/.

[35] Benjamini Y., Hochberg Y. (1995). Controlling the false discovery rate: A practical and powerful approach to multiple testing. J. R. Stat. Soc. Ser. Methodol..

[36] Benjamini Y., Yekutieli D. (2001). The control of the false discovery rate in multiple testing under dependency. Ann. Stat..

[37] Benjamini Y., Yekutieli D. (2005). False discovery rate—Adjusted multiple confidence intervals for selected parameters. J. Am. Stat. Assoc..

[38] David Groppe (2020). fdr_bh. MATLAB Central File Exchange. https://www.mathworks.com/matlabcentral/fileexchange/27418-fdr_bh).

[39] Tibshirani R. (1996). Regression shrinkage and selection via the lasso. J. R. Stat. Soc. Ser. Methodol..

[40] Balconi M., Brambilla E., Falbo L. (2009). BIS/BAS, cortical oscillations and coherence in response to emotional cues. Brain Res. Bull..

[41] Balconi M., Mazzà G. (2009). Brain oscillations and BIS/BAS (behavioral inhibition/activation system) effects on processing masked emotional cues. Int. J. Psychophysiol..

[42] Klados M., Frantzidis C., Vivas A.B., Papadelis C., Lithari C., Pappas C., Bamidis P.D. (2009). A framework combining delta Event-Related Oscillations (EROs) and synchronisation effects (ERD/ERS) to study emotional processing. Comput. Intell. Neurosci..

[43] Aftanas L.I., Varlamov A.A., Pavlov S.V., Makhnev V.P., Reva N.V. (2002). Time-dependent cortical asymmetries induced by emotional arousal: EEG analysis of event-related synchronization and desynchronization in individually defined frequency bands. Int. J. Psychophysiol..

[44] Balconi M., Brambilla E., Falbo L. (2009). Appetitive vs. defensive responses to emotional cues. Autonomic measures and brain oscillation modulation. Brain Res..

[45] Westphal K.P., Grözinger B., Diekmann V., Scherb W., Rees J., Leibing U., Kornhuber H.H. (1990). Slower theta activity over the midfrontal cortex in schizophrenic patients. Acta Psychiatr. Scand..

[46] Aftanas L.I., Varlamov A., Pavlov S., Makhnev V., Reva N. (2001). Affective picture processing: Event-related synchronization within individually defined human theta band is modulated by valence dimension. Neurosci. Lett..

[47] Başar E., Güntekin B., Oniz A. (2006). Principles of oscillatory brain dynamics and a treatise of recognition of faces and facial expressions. Sleep Deprivation Cogn..

[48] Sun J., Sun B., Wang B., Gong H. (2012). The processing bias for threatening cues revealed by event-related potential and event-related oscillation analyses. Neurosci..

[49] Güntekin B., Başar E. (2007). Emotional face expressions are differentiated with brain oscillations. Int. J. Psychophysiol..

[50] Güntekin B., Başar E. (2007). Gender differences influence brain’s beta oscillatory responses in recognition of facial expressions. Neurosci. Lett..

[51] Engel A.K., Fries P. (2010). Beta-band oscillations—Signalling the status quo?. Curr. Opin. Neurobiol..

[52] Martini N., Menicucci D., Sebastiani L., Bedini R., Pingitore A., Vanello N., Milanesi M., Landini L., Gemignani A. (2012). The dynamics of EEG gamma responses to unpleasant visual stimuli: From local activity to functional connectivity. NeuroImage.

[53] Müller M.M., Keil A., Gruber T., Elbert T. (1999). Processing of affective pictures modulates right-hemispheric gamma band EEG activity. Clin. Neurophysiol..

[54] Rouw R., Kosslyn S.M., Hamel R. (1997). Detecting high-level and low-level properties in visual images and visual percepts. Cognition.

[55] Miskovic V., Martinovic J., Wieser M., Petro N.M., Bradley M.M., Keil A. (2015). Electrocortical amplification for emotionally arousing natural scenes: The contribution of luminance and chromatic visual channels. Biol. Psychol..

[56] Schindler S., Schettino A., Pourtois G. (2018). Electrophysiological correlates of the interplay between low-level visual features and emotional content during word reading. Sci. Rep..

