# Supplementary material for: The Temporal and Spatial Dynamics of Cortical Emotion Processing in Different Brain Frequencies as Assessed Using the Cluster-Based Permutation Test: An MEG Study
Source: Brain Sci. 2020 Jun 6;10(6):352. doi: 10.3390/brainsci10060352 (PMC7349493; doi:10.3390/brainsci10060352)
Supplement: Supplementary file 1 [file brainsci-10-00352-s001.pdf]

brain sciences

MDPI

Supplementary materials

1. The position of MEG channels

To determine which brain areas are related to the MEG channels selected by CBPT in our results, we referred to Figure S1 [1, 2], which shows Elekta Neuromag MEG channel groupings in different brain areas.

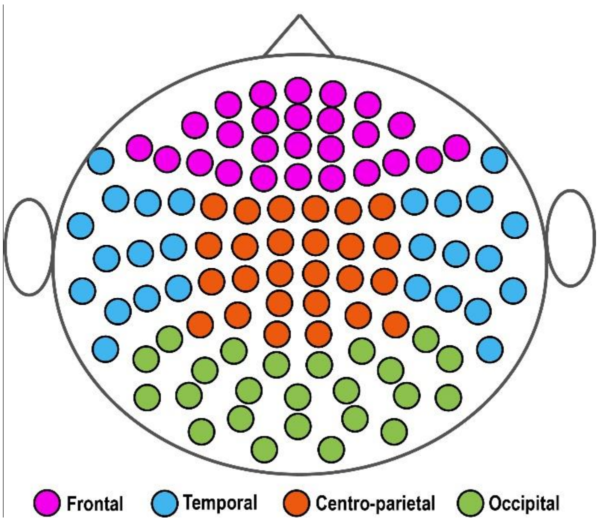

Figure S1. Presentation of the MEG sensor grouping in five brain areas

2. Assessing the stimuli

After the MEG measurement, subjects were requested to rate each picture stimuli using the Self-Assessment Manikin (SAM; [3]). SAM provides a seven-point scale indicating arousal (1 to 7, relaxed to excited) and valence (1 to 7, pleasant to unpleasant) levels. Comparing the mean rating of subjects for each picture category using the t-test we found significant differences which are shown in Figure S2 and Table S1.

Table S1. Results of comparing mean ratings of subjects for each picture category in this study.

	Comparison	p-value
Valence	Pl vs. Ne	10^{-29}
	Un vs. Ne	10^{-38}
	Un vs. Pl	10^{-50}
Arousal	Pl vs. Ne	10^{-26}
	Un vs. Ne	10^{-37}
	Un vs. Pl	10^{-19}

Pl: Pleasant, Ne: Neutral, Un: Unpleasant.

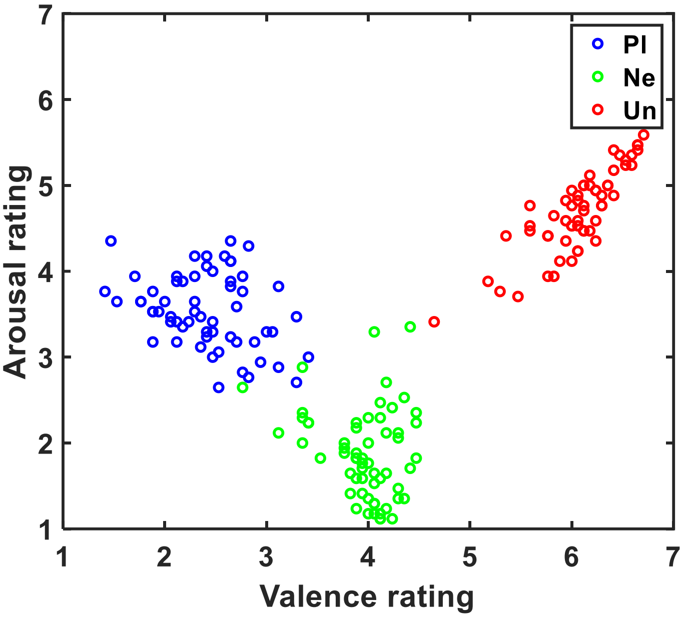

Figure S2. Mean rating of arousal and valence over subjects for each picture category. Circles show the mean ratings of arousal and valence across all subjects for each picture category (pleasant (Pl): blue circles; neutral (Ne): green circles; unpleasant (Un): red circles) selected from the International Affective Picture System (IAPS). The horizontal axis shows valence (pleasant-to-unpleasant) values and the vertical axis shows arousal (low-to-high) values from a 1-7 scale. Unpleasant pictures were rated as the most arousing pictures compared to pleasant and neutral stimuli. Pleasant stimuli were rated as the lowest valence pictures and unpleasant stimuli as the highest valence pictures.

3. Classification method

We performed a classification algorithm on features based on the selected channels and time-intervals by the CBPT. We took the mean values of the power spectrum over all trials of each emotion category and over frequencies of a particular frequency-band and over the selected time-windows by CBPT as observations for each subject. Thus in each frequency-band, each subject provided three vector-valued observations for each frequency-band (5 bands): one for pleasant, one for neutral, and one for unpleasant. Each observation in each comparison incorporates a specific number of elements which were the magnetometers selected by CBPT. Therefore, for both two comparisons (pleasant and unpleasant vs. neutral) we compiled special feature sets based on selected channels and selected time-windows from CBPT with a dimensionally of 34 samples (17 subjects × 2 conditions) × number of selected channels by CBPT in each time-windows.

After feature sets definition, we applied the regularized logistic regression with the most popular penalty, the Least Absolute Shrinkage and Selection Operator (LASSO; [4]), to the feature-sets. LASSO is a highly common classification method and because it selects the most reliable feature-subsets by forcing some regression coefficients to zero, it provides feature-subset selection together with high classification accuracies [5]. Here, we defined the response variable of

the logistic regression by one for emotional conditions (pleasant and unpleasant), and zero for the neutral condition. Accordingly, the probability of being in an emotional state (class 1) for the kth subject is estimated by Equation (1):

$$\pi_k = p(y_k = 1|x_k) = \frac{\exp\ (\beta_0+\sum_{m=1}^{M} x_{km}{}^T\beta_m)}{1\ +\ \exp\ (\beta_0+\sum_{m=1}^{M} x_{km}{}^T\beta_m)} \qquad k = 1, 2, \ldots, K \tag{1}$$

where $\mathrm{y_k} \in \{0, 1\}$ is a vector with *k* elements, x_k are associate vectors with *M* predictors, β_m are the regression coefficients, and β_0 is the intercept. The ratio of the probability of class 1 divided by the probability of the class 0 (which is called odds function) [6] and then logit transformation is estimated by Equation (2):

$$Odds = \frac{\pi_k}{1-\pi_k} = \exp\ (\beta_0 + \sum_{m=1}^{M} \boldsymbol{x}_{km}{}^T\beta_m) \Rightarrow Ln\left(\frac{\pi_k}{1-\pi_k}\right) = \beta_0 + \sum_{m=1}^{M} \boldsymbol{x}_{km}{}^T\beta_m \tag{2}$$

To estimate the β_m and β_0 we have to calculate log-likelihood function, which is defined as Equation (3):

$$l(\ \beta_0, \beta) = \sum_{k=1}^{K} \{y_k \ln(\pi_k) + (1 - y_k) \ln(1 - \pi_k)\} \tag{3}$$

which estimates the regression coefficients β_m based on both test and train data and leads to lower variances and better classification accuracies. The tuning parameter, λ, controls the shrinkage. This means When $\lambda = 0$, the penalty term has no effect, and as $\lambda \rightarrow \infty$, the impact of the penalty term grows. However, to minimizing the whole equation, the regression coefficients will approach zero in the case of $\lambda \rightarrow \infty$. This forces some of the coefficients to become zero. Thus, selecting the best value for λ is critical, and this is usually done using cross-validation, which involves testing a number of different values and selecting one that minimizes cross-validated error on test data [6, 7]. In this study, we performed leave-one-subject-out cross-validation to selected optimal λ for each feature sets. Then the classification performances using this λ for each feature sets were evaluated by accuracy (the ratio of correctly classified samples to their total number). The accuracies were also assessed performing 1000 17-fold-stratified cross-validations and 95%- confidence intervals of them were reported. It should be noted that we used 17 folds because we had 17 subjects.

References

1. Abadi, M.K.; Subramanian, R.; Kia, S.M.; Avesani, P.; Patras, I.; Sebe, N. DECAF: MEG-based multimodal database for decoding affective physiological responses. *IEEE Transactions on Affective Computing* **2015**, 3, 209-222, doi:10.1109/TAFFC.2015.2392932.
2. Hauk, O. *Vectorview Description - Meg Wiki, Elekta Neuromag Vectorview Syst*. **2013**; Available from: http://imaging.mrc-cbu.cam.ac.uk/meg/VectorviewDescription.
3. Lang, P.J. Behavioral treatment and bio-behavioral assessment: Computer applications. *Technol. Ment. Health care Deliv. Syst.* **1980**, 119-137.

4. Tibshirani, R. Regression shrinkage and selection via the lasso. J. R. Stat. Soc. Ser. B (Methodol.) 1996, 58, 267–288.
5. Algamal, Z.Y.; Lee, M.H. Penalized logistic regression with the adaptive LASSO for gene selection in high-dimensional cancer classification. Expert Syst. Appl. 2015, 42, 9326–9332.
6. James, G., Witten, D., Hastie, T. and Tibshirani, R., 2013. An introduction to statistical learning (Vol. 112, p. 18). New York: springer.
7. Czepiel, S.A., 2002. Maximum likelihood estimation of logistic regression models: theory and implementation. Available at czep. net/stat/mlelr. pdf.
